# Genomic and phenotypic studies among *Clostridioides difficile* isolates show a high prevalence of clade 2 and great diversity in clinical isolates from Mexican adults and children with healthcare-associated diarrhea

**DOI:** 10.1128/spectrum.03947-23

**Published:** 2024-06-12

**Authors:** D. Meléndez-Sánchez, Laura Hernández, Miguel Ares, A. Méndez Tenorio, Lourdes Flores-Luna, Javier Torres, M. Camorlinga-Ponce

**Affiliations:** 1 Posgrado en Biomedicina y Biotecnología Molecular, Escuela Nacional de Ciencias Biológicas, Instituto Politécnico Nacional, Ciudad de México, México; 2 Human Systems Biology Laboratory, Instituto Nacional de Medicina Genómica (INMEGEN), México City, México; 3 Unidad de Investigación en Enfermedades Infecciosas y Parasitarias, UMAE Pediatría, Instituto Mexicano del Seguro Social, México City, México; 4 Laboratorio de Bioinformática y Biotecnología Genómica, Departamento de Bioquímica, Escuela Nacional de Ciencias Biológicas, Instituto Politécnico Nacional, México City, México; 5 Centro de Investigación en Salud Poblacional, Instituto Nacional de Salud Pública, Cuernavaca, Morelos, México; Johns Hopkins University, Baltimore, Maryland, USA

**Keywords:** *Clostridioides difficile*, toxin gene expression, whole-genome sequence, clade, sequence type (ST), toxin subtype

## Abstract

*Clostridioides difficile* (*C. difficile*) is widely distributed in the intestinal tract of humans, animals, and in the environment. It is the most common cause of diarrhea associated with the use of antimicrobials in humans and among the most common healthcare-associated infections worldwide. Its pathogenesis is mainly due to the production of toxin A (TcdA), toxin B (TcdB), and a binary toxin (CDT), whose genetic variants may be associated with disease severity. We studied genetic diversity in 39 *C*. *difficile* isolates from adults and children attended at two Mexican hospitals, using different gene and genome typing methods and investigated their association with *in vitro* expression of toxins. Whole-genome sequencing in 39 toxigenic *C. difficile* isolates were used for multilocus sequence typing, *tcdA*, and *tcdB* typing sequence type, and phylogenetic analysis. Strains were grown in broth media, and expression of toxin genes was measured by real-time PCR and cytotoxicity in cell-culture assays. Clustering of strains by genome-wide phylogeny matched clade classification, forming different subclusters within each clade. The toxin profile *tcdA+/tcdB+/cdt+* and clade 2/ST1 were the most prevalent among isolates from children and adults. Isolates presented two TcdA and three TcdB subtypes, of which TcdA2 and TcdB2 were more prevalent. Prevalent clades and toxin subtypes in strains from children differed from those in adult strains. Toxin gene expression or cytotoxicity was not associated with genotyping or toxin subtypes. In conclusion, genomic and phenotypic analysis shows high diversity among *C. difficile* isolates from patients with healthcare-associated diarrhea.

**IMPORTANCE:**

*Clostridioides difficile* is a toxin-producing bacterial pathogen recognized as the most common cause of diarrhea acquired primarily in healthcare settings. This bacterial species is diverse; its global population has been divided into five different clades using multilocus sequence typing, and strains may express different toxin subtypes that may be related to the clades and, importantly, to the severity and progression of disease. Genotyping of children strains differed from adults suggesting toxins might present a reduced toxicity. We studied extensively cytotoxicity, expression of toxins, whole genome phylogeny, and toxin typing in clinical *C. difficile* isolates. Most isolates presented a *tcdA+/ tcdB+/cdt*+ pattern, with high diversity in cytotoxicity and clade 2/ST1 was the most prevalent. However, they all had the same TcdA2/TcdB2 toxin subtype. Advances in genomics and bioinformatics tools offer the opportunity to understand the virulence of *C. difficile* better and find markers for better clinical use

## INTRODUCTION

*Clostridioides difficile* is currently considered one of the most important causes of healthcare-associated diarrhea, although the number of community-based cases is increasing (1). *C. difficile* is a Gram-positive, anaerobic, spore-forming bacillus widely distributed in the intestinal tract of humans, animals, and in the environment (2). Shortly after the identification of hypervirulent *C. difficile* strains RT027 (B1/NAP1/ST1) in Canada, there were reports of CDI outbreaks caused by this strain in the United States and Europe and then Asia, Australia, and Latin America (3). In Mexico *C. difficile* RT027 strain has been confirmed in hospitalized patients (4–6).

The clinical outcome of *C. difficile* infection (CDI) ranges from mild diarrhea to life-threatening pseudomembranous colitis, where virulence is mainly driven by the action of toxin A (TcdA) and toxin B (TcdB). TcdA and TcdB are 308 and 270 kDa proteins, respectively, belonging to a large Clostridial toxin family that glycosylate Rho family GTPases in host cells, leading to the disruption of the actin cytoskeleton, cell death, and a strong inflammatory response (7, 8). The genes that code for TcdA (*tcdA*) and TcdB (*tcdB*) are located within a 19.6 kb chromosomal region called the pathogenicity locus (PaLoc) (9, 10). PaLoc contains five genes, two of which code for TcdA and TcdB, and three additional genes that are involved in the regulation, production, and secretion of toxins (*tcdR*, *tcdC*, and *tcdE*) (9, 10). Approximately 20% of *C. difficile* strains produce a third toxin called *C. difficile* transferase (CDT) or binary toxin, which is an ADP-ribosyltransferase encoded in a locus defined as the CdtLoc (11). CDT consists of the *cdtA* and *cdtB* genes encoding the two subunits of CDT, plus *cdtR*, which encodes a positive regulatory protein (12).

Studies carried out with strains mutant in *tcdA* and *tcdB* in different animal models, along with the isolation of strains that produce only TcdB, have helped define that TcdA may contribute to disease severity, although TcdB alone can induce the full spectrum of disease in both animals and humans (13, 14). *C. difficile* strains may encode different patterns of toxins, coding for one, two, three, or none of the toxins, primarily due to recombination and their highly mobile genome (15). Sequence analysis has revealed the existence of TcdA and TcdB variants (13, 16) that are associated with biological properties such as enzymatic activity, immunogenicity, and affinity for receptors, suggesting variants may also be related to the severity of the disease (13, 16, 17). Studies have identified 12 TcdB subtypes/variants (13), and strains like *C. difficile* 630 may present different TcdB variants, TcdB2, TcdB3, and TcdB4 (13, 18, 19) and up to 7 subtypes/variants for TcdA (13).

In recent years, significant advances have been made in our understanding of the pathogenicity of *C. difficile* because of the identification and molecular characterization of the major toxins TcdA and TcdB. However, few studies have focused on the association of the pattern of coding toxin genes and toxins subtypes with expression levels and cytotoxicity of TcdA, TcdB, and CDT. The possible association of genotyping of the strains [multilocus sequence typing (MLST), clade, sequence types (STs), and whole-genome phylogeny] with toxin expression or cytotoxicity has not been studied either (9, 13). In addition, there is limited information on the subtypes of toxins in clinical isolates from pediatric patients. The aim of our study was to analyze the expression and typing of *tcdA*, *tcdB*, and *cdt* genes, and the genotyping of 39 *C. difficile* strains isolated from adult and pediatric patients from two Mexican hospitals. For genotyping, MLST, clades, ST, and whole-genome phylogeny were determined and studied for any association with coding patterns, levels of expression, and variants of the *tcdA* and *tcdB* toxin genes.

## RESULTS

### Characterization of patients and amplification of toxin genes

Table 1 summarizes the demographic and clinical information of the patients, risk factors, and the toxin gene profiles of the *C. difficile* strains analyzed. Thirty-nine toxigenic *C. difficile* strains from patients who attended two Mexican hospitals were included in this study; of these, 31 were isolated from adults and 8 from children. All 39 patients had co-morbidities and some significant differences were observed between adults and children (*P* = 0.001). Gastrointestinal diseases were the most frequent co-morbidity in children (*P* = 0.001), while kidney and cardiovascular diseases were in adults. The use of antibiotics before diarrhea was also statistically significant (*P* = 0.056). The most commonly used antibiotics were cephalosporins, quinolones, penicillins, and vancomycin in adults, while in children metronidazole, aminoglycosides, and vancomycin were the most common.

**TABLE 1:** Characteristics of *Clostridioides difficile* strains isolated from children and adults with healthcare-associated diarrhea in Mexico

	Adults *n* = 31 No. (%)	Children *n* = 8 No. (%)	Total *n* = 39 No. (%)
Age, mean ± SD	57.4 ± 14.7	6.2 ± 6.0	35.2 ± 25.0
Gender			
Male	14 (45.1)	5 (62.5)	19 (48.7)
Female	17 (54.9)	3 (37.5)	20 (51.2)
Co-morbid conditions			
Gastrointestinal	6 (19.3)	6 (75)	12 (30.7)^*a*^
Malignancy	0.0	2 (25)	2 (5.1)
Hematologic	3 (9.6)	0.0	3 (7.6)
Renal	8 (25.8)	0.0	8 (20.5)
Cardiovascular	8 (25.8)	0.0	8 (20.5)
Metabolic	6 (19.3)	0.0	6 (15.3)
Potential risk factors			
Antibiotics use before diarrhea	30 (96.7)	6 (75)	36 (92.3)
Hospitalization days before diarrhea (Median, range)	5.5 (3–60)	20 (3–80)	
Toxin genes profile			
*tcdA+/tcdB+/cdtA+/cdtB+*	28 (93.5)	5 (62.5)	33 (84.6)^*b*^
*tcdA−/tcdB+/cdtA−/cdtB−*	2 (6.5)	0.0	2 (5.1)
*tcdA+/tcdB+/cdtA−/cdtB−*	1 (3.2)	2 (25.0)	3 (7.7)
*tcdA−/tcdB−/cdtA−/cdtB+*	0.0	1 (12.5)	1 (2.5)

^*a*^ Fisher’s exact test, *P* = 0.001.

^*b*^ Test of proportions, *P* < 0.05, the proportion *tcdA+tcdB+cdtA+cdtB*+ is different between adults and children strains.

Thirty-three of the 39 *C*. *difficile* isolates (84.6%) were positive for all three toxin genes (*tcdA+/tcdB+*/*cdt+*). Other toxin profiles were less frequent: two (5.1%) were *tcdA−/tcdB+/cdt*−, three (7.7%) were *tcdA+/tcdB+/cdt*−, and one isolate from a child was *tcdA−/tcdB−/cdt+*. The toxin pattern *tcdA+/tcdB+/cdt+* was significantly more frequent in isolates from adults than in isolates from pediatric patients (*P* < 0.05).

### Production of toxins *in vitro*

**Fig 1:**
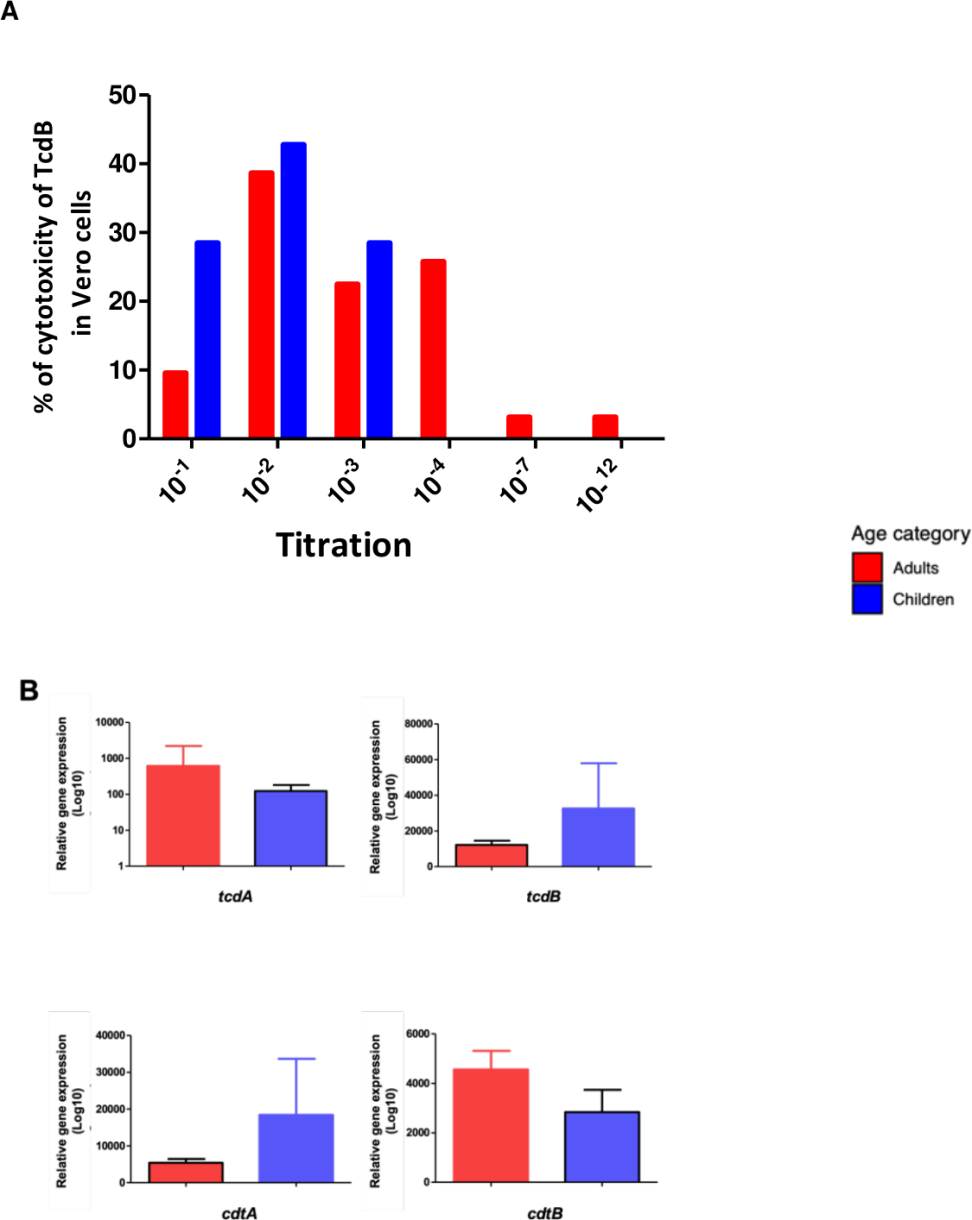
Cytotoxicity of *C. difficile* TcdB and analyses of the expression of toxins *tcdA, tcdB, cdtA, and cdtB*. (**A**) Titration of cytotoxicity of *C. difficile* TcdB on Vero cells after 24 h incubation (*P* = 0.46). (**B**) Level of expression of toxin genes *tcdA, tcdB, cdtA*, and *cdtB*, the mean and standard deviation from triplicates are shown; no significant difference was found. Each toxin gene expression was normalized using *rpoA* expression as a reference.

Once we learned about the toxin gene profiles, we asked whether there was any difference in the cytotoxic activity of strains after *in vitro* culture. As shown in Fig. 1A, the supernatant of the adult *C. difficile* strains showed higher cytotoxicity titers than the isolates from children; however, the results were not statistically significant (*P* = 0.46) probably due to the sample size (Fig. 1A).

### Toxin gene expression

Differences were observed in the relative expression of toxin genes by strains isolated from adults versus children (Fig. 1B); whereas adults expressed more *tcdA* than children did, and children expressed more *tcdB* than adults (Fig. 1B). Similarly, whereas *cdtA* was expressed more in children, *cdtB* was more expressed in adults (Fig. 1B). In spite of the observed tendency for differences in toxins expression between children and adult strains, none of them resulted significantly (*P* > 0.05). Still, the relative expression *tcdA/tcdB* was 4.9 in adults and 0.37 in children’s strains. In contrast, the relative expression of *cdtA/cdtB* was 0.29 in adults and 1.29 in children’s strains.

### Subtyping of TcdA and TcdB

**Fig 2:**
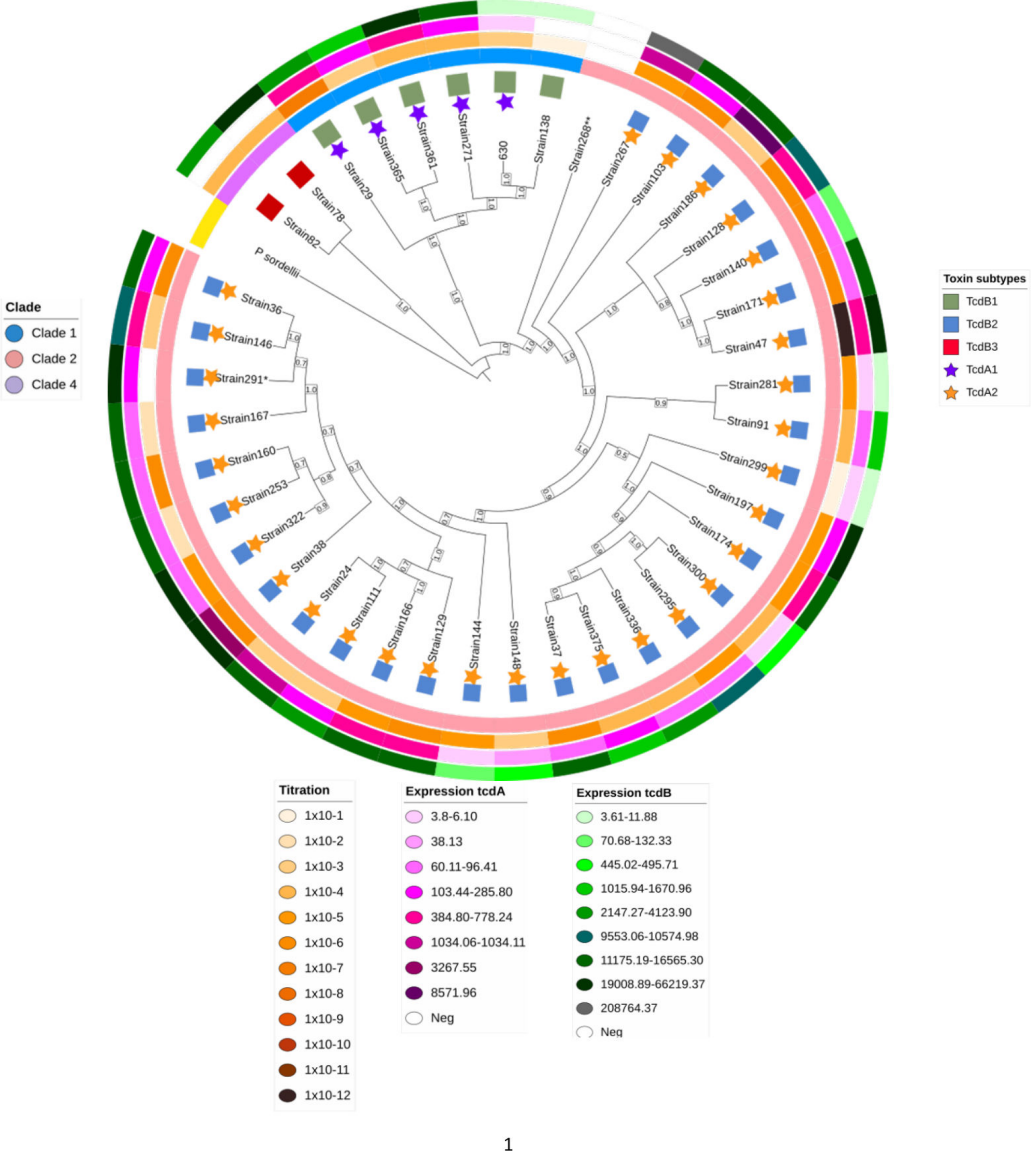
Phylogenetic analysis of TcdA and TcdB subtypes of 39 *C*. *difficile* isolates from Mexican patients. The phylogenetic tree illustrates the relationship between subtype, clade, cytotoxicity, and expression of *tcdA* and *tcdB* toxins in each isolate. Toxin subtype is indicated on the side of the strain number, with a star for TcdA and a square for TcdB. From inside-out, the first ring shows the clade for each strain. The second ring shows the cytotoxicity titer. The third ring indicates the *tcdA* expression, and the fourth ring shows *tcdB* expression. Color code values for each variable are indicated around the tree. Strain 268*** had the toxin pattern: *tcdA−/tcdB*−. TcsH hemorrhagic toxin from *P. sordelli* was used as an outgroup. The bootstrap test with 250 replicates was used to assess confidence of the phylogenetic analysis and values are indicated in the branches of the tree.

The 39-genome sequences from *C. difficile* clinical isolates were analyzed for TcdA and TcdB toxin subtypes. We found two TcdA subtypes and three TcdB subtypes in the Mexican isolates (Table 3). In the analyses of TcdA, most of the strains (67.5%) had a TcdA2.1 subtype. Analysis of TcdB subtypes showed that TcdB2.1 was the predominant subtype (76.3%), although other subtypes were also identified. The subtypeTcdA2.1/TcdB2.1 was the dominant toxins subtype. Also, we examined the relationship between TcdA and TcdB subtypes and the *C. difficile* whole-genome phylogeny and observed a strong correlation between the TcdA subtype and clades (*r* = 0.86; *P* < 0.001) and moderate for subtypes and clades of TcdB (*r* = 0.32; *P* = 0.05). All clade 2 were TcdA2/TcdB2, four clade 1 were TcdA1/TcdB1, and two clade 4 were TcdA1/TcdB3 (Fig. 2).

### Genotyping analysis

**Fig 3:**
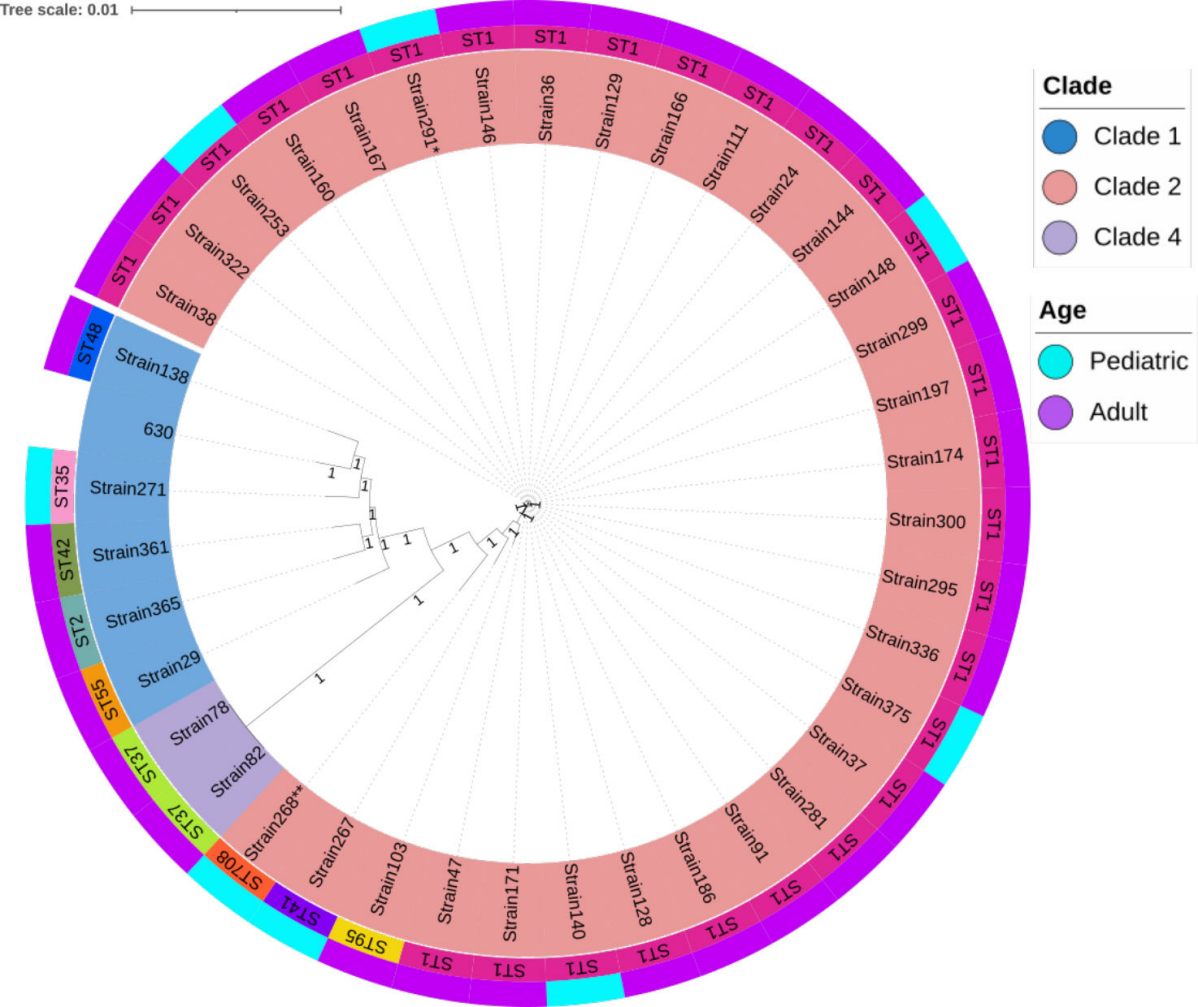
Phylogenetic analysis of 39 *C*. *difficile* isolates using single nucleotide polymorphisms (SNPs) in the core genome. The phylogenetic tree illustrates the relationships of clades (inner circle), STs (middle circle), and age groups (outer circle) of the *C. difficile* isolates. *C. difficile* strain 630 was used as control. The bootstrap test with 250 replicates was used to assess confidence in the phylogenetic analysis, and values are indicated in each branch of the tree.

The MLST analyses of the 39 *C*. *difficile* genome sequences showed the presence of clades 1, 2, and 4 and 10 different STs (Table 2; Fig. 3). Thirty-three (85%) isolates were clade 2, and 29 of them were ST1 (74% of the 39 strains were clade 2/ST1). All other ST types were present in a single strain, except for ST37, present in two adult isolates with the pattern (*tcdA−/tcdB+/cdt*−) that belonged to clade 4, whereas the single strain (*tcdA−/tcdB−/cdtA−/cdtB+*) was isolated from a child and was clade 2.

**TABLE 2:** Distribution of toxin profile, clades, and sequence type, in 39 *Clostridioides difficile* strains isolated from adults and children in Mexico

Toxins profile	Clade	Sequence type (ST)	Adults *n* = 31 No. (%)	Children *n* = 8 No. (%)
*tcdA+/tcdB+/cdtA+/cdtB+*				
	2	1	24 (77.4)	5 (62.5)
	1	2	1 (3.2)	0.0
	1	42	1 (3.2)	0.0
	2	48	1 (3.2)	0.0
	2	95	1 (3.2)	0.0
*tcdA−/tcdB+/cdtA−/cdtB−*				
	4	37	2 (6.4)	0.0
*tcdA+/tcdB+/cdtA−/cdtB−*				
	1	35	0.0	1 (12.5)
	2	41	0.0	1 (12.5)
	1	55	1 (3.2)	0.0
*tcdA−/tcdB−/cdtA−/cdtB+*				
	2	708	0.0	1 (12.5)

### Genome phylogenetic analysis

A phylogenetic analysis was performed with the whole-genome sequences of the 39 *C*. *difficile* strains. SNPs from the *C. difficile* core genome were analyzed to estimate the evolutionary divergence between the sequences. The tree was divided into three main branches, each consisting of strains belonging to clades 2, 1, or 4 (Fig. 3). The bootstrap value for most branches in all clades was 1, confirming the reliability of the phylogenetic analyses. In some cases, unusual STs were associated with subgroups such as ST708, ST41, and ST95 within the large group of clade 2. Different types of ST were observed among the strains included in clade 1. Genome-wide analyses did not distinguish between isolates from adults and children, although unusual ST types were more common in strains from children (Fig. 3).

### Phylogenetic analyses with concatenated TcdA and TcdB amino acid sequences

We next asked if there titers, was any association between the phylogeny of TcdA and TcdB genotypes with clades, ST, cytotoxicity, or gene expression (Fig. 2). Phylogeny of TcdA and TcdB showed a clear clustering according to clades; thus, all clade 2 strains were TcdA2/TcdB2, except one nontoxigenic (strain 268). Also, all clade 1 strains were TcdA1/TcdB1, and those clade 4 were TcdA1/TcdB3. Still, even within each clade and toxin genotype, there was sequence diversity in the toxin genes as evidenced in the tree (Fig. 2). Thus, whereas 25 of the 31 clade 2-TcdA2/TcdB2 strains fell into a subcluster with almost no distance between them (Fig. 3, from Strain 38 to Strain 268) the other six were in separated clusters, three were ST1 type, whereas the other three showed uncommon ST’s (08, 41, and 95). It is of interest to note that three of these six strains were isolated from children. In addition, in clades 4 and 1, the toxins sequence was diverse among all strains. Thus, whereas clades are strongly correlated with toxin types, sequences of TcdA and TcdB present diversity. No correlation of toxins genotype with cytotoxicity or toxin gene expression was observed (Fig. 2). There was a high diversity in cytotoxicity and expression of genes, even within the 28 strains clade 2-ST1-TcdA2/TcdB2, with a range from Strain 299 with 10^−1^ cytotoxicity/<6.0 *tcdA* expression/<10 *tcdB* expression to Strain 47 with 10^−12^ cytotoxicity/500 *tcdA* expression/>20,000 *tcdB* expression. Also, cytotoxicity and toxin expression were variable among the six clade 1-TcdA1/TcdB1 strains. The bootstrap value is between 0.05 and 1.0, reflecting the reliability of obtained phylogenetic estimation.

## DISCUSSION

During the 80s–90s, we learned about the main role of TcdA and TcdB in the pathogenesis of *C. difficile* infection, but little was known about the molecular epidemiology of the infection. At that time, strains coding for only one toxin (*tcdA−/tcdB*+) were very rare (20), but thanks to updated genomic techniques, we are learning more about the molecular epidemiology of the infection, and know that *C. difficile* clade 2-ST1 strain has spread throughout the world, including Latin America (1), although in recent years the Incidence of CDI, is decreasing (21, 22). However, the epidemic keeps evolving, and new clades have emerged with a wide diversity of genotypes in different countries (clades 1, 2, 3, 4, and 5 and tree cryptic clades have been described) (23, 24). Studies have shown that clade 1 is more prevalent worldwide. Thus, clade 1, ST04, and ST037 strains were reported as the most frequent in Israel (25), whereas in Mexico, we found clade 2-ST1 strains as the more prevalent (6). Furthermore, we found clades 4, 2 and 1 strains with unusual STs (37, 35, 48, 55, 95, and ST708) and some of these STs have been isolated from different environmental, animal, and clinical sources (26) but are now circulating among humans and emerging as clinically important strains.

*C.difficile* isolates of the molecular type clade 2-ST1 have been associated with severe disease and hospital outbreaks, and it has been suggested that these “hypervirulent” strains produce higher levels of TcdA and TcdB toxins besides the CDT toxin (27). In the present work, we did a comprehensive characterization of the clade2-ST1 prevalent strains, including cytotoxicity, toxins profile, toxins expression, and whole-genome phylogeny.

Most of these clade 2-ST1 strains presented the toxins profile *tcdA+/tcdB+/cdtA+*; still, they showed a high diversity in cytotoxicity and expression of both *tcdA* and *tcdB*, although they all had the same toxins subtype TcdA2/TcdB2. Watanabe et al. also found no association between clades and toxin gene expression (28). In agreement with our findings, recent evidence suggests that clade 2-ST1 isolates do not produce more toxins *in vitro* than isolates from other lineages (28), as was initially reported for hypervirulent *C. difficile* RT027 (29).

Evidence of high homologous recombination in the *tcdB* gene have been found, suggesting a strong positive selective pressure, and resulting in diversification of the gene sequence (16). These findings are consistent with our results and may explain the number of subtypes that we identified in the analyzed sequences (Table 3; Fig. 3), three subtypes in TcdB and two subtypes in TcdA.

**TABLE 3:** Distribution of TcdA and TcdB subtypes of Mexican *Clostridioides difficile* strains

Subtype A^*a*^	Strains No. (%)	Subtype B^*a*^	Strains No. (%)
A1.1	3 (8.1)	B1.2	2 (5.3)
A1.6	2 (5.4)	B1.3	1 (2.6)
A1.8	1 (2.7)	B1.4	1 (2.6)
A2.1	25 (67.5)	B2.1	29 (76.3)
A2.14	3 (8.1)	B2.19	2 (5.3)
A2.3	1 (2.7)	B2.22	1 (2.6)
A2.4	2 (5.7)	B3.1	2 (5.3)
Total	37	Total	38

^*a*^ Fisher’s exact test between subtypes A and B (*P* < 0.001).

It was interesting to observe that the SNPs in the whole-genome phylogenic analyses clearly grouped clade 2-ST1 strains into different clusters (Fig. 3), suggesting the presence of differences in other regions of the genome that may also influence virulence, like outer membrane proteins or sporulation genes (12). A deeper analysis of the genomes may identify genes or regions responsible for differentiating the clades, and to better understand the virulence of the strains, which in turn may be useful for the development of efficient test for molecular epidemiology studies.

We also report differences in the genotyping and expression of toxins in between isolates from children and adults, whose possible differences are poorly described. Although the number of children strains was low, some differences were observed. The expression levels of *tcdA*, *tcdB*, *cdtA*, and *cdtB* were markedly different compared to adult isolates (Fig. 1). In addition, the presence of rare STs was common in children isolates (ST41, 708, and 35), and the only strain clade 2 that had no *cdtA* and *cdtB* genes was from a child (Strain 268, clade 2-ST708). Although these results need to be confirmed, our findings strongly suggest that the epidemiology of CDI in children might differ from that in adults, which may result in differences in virulence and transmission.

We identified three subtypes for TcdB and two subtypes for TcdA as prevalent in our community, although additional subtypes might be identified when studying a larger number of strains, as reported by Shen et al. after examining strains from different geographic regions (13). TcdB2 was the subtype we most frequently identified, unlike studies in other geographical areas where TcdB1 was the most common (13, 16). The study of toxin subtype is relevant, whereas initial studies reported that TcdB1 strains might be more cytotoxic, recent analyses suggest that TcdB2 is more cytotoxic in other populations (19). Furthermore, it has been reported that sequence variations between subtypes may have an impact on therapeutic treatment with antibodies and vaccines (16). Clearly, more studies on toxins typing, virulence, and treatment are needed. We observed that, for example, not all *tcdA*2/*tcdB*2 are homologous, and different clusters are observed (Fig. 2) that may have implications on the pathogenic activity. To test the toxicity of B subtypes, Shen et al., injected toxins intraperitoneally into mice and observed that TcdB1, TcdB2, and TcdB3 are more toxic compared to the other subtypes (13). TcdB targets the colonic epithelium by binding to frizzled (FZD); however, TcdB2 has been shown to weakly bind to FZD compared to TcdB1 (21), probably due to discrepancies in the FZD binding sequences of TcdB1 and TcdB2 (30). A clear limitation of this study is the low number of isolates tested, which reduces the reach of our findings, particularly in strains isolated from children. Still, the genomic and phenotypic results of our work offer clues on the diversity of *C. difficile* strains circulating in one community and document the presence of uncommon genotypes (clades and ST) in Mexico. To our knowledge, this study reports for the first time genomic and virulence properties in pediatric strains. Also, no clear correlation was found between genotypic and phenotypic properties, for example, the cytotoxicity and expression of toxin genes varied even within clade 2-ST1 strains, and no evidence of uniform high toxicity was revealed, as previously suggested. This finding has important clinical implications for physicians and microbiologists.

During the first pandemic, strains were classified as toxigenic or non-toxigenic and it was thought that *C. difficile* was more of a clonal species. In this second pandemic, advances in genomics and bioinformatics tools offer the opportunity to go further and extend our studies to all genome content to understand virulence better and to eventually find markers with better clinical use, particularly in Latin America where studies on *C. difficile* infection are badly needed.

## MATERIALS AND METHODS

### Patients and isolation of *Clostridioides difficile*

A total of 39 *C*. *difficile* strains isolated from patients with hospital-acquired diarrhea were analyzed, 31 isolated from adults with an age ranging from 20 to 82 years (mean 57.4 ± 14.7 years) and eight from children with an age from 1 to 15 years (mean 6.2 ± 6.0 years). Consecutive patients were recruited from two hospitals (one pediatric and one general hospital) at the Centro Médico Nacional Siglo XXI, Instituto Mexicano del Seguro Social in Mexico City from 2016 to 2018. The ethical committee from the Instituto Mexicano del Seguro Social approved the study, with the number: R-2015-785-089 and patients or their guardians were informed about the study and asked to sign an informed consent for participation.

To isolate *C. difficile*, stool samples were treated with 96% ethanol at room temperature for 50 min, followed by centrifugation at 4000 rpm for 10 min (6). The pellets were inoculated into taurocholate-cycloserine-cefoxitin-fructose agar (CCFA) and incubated at 37°C for 48 h under anaerobic conditions in a jar with an atmosphere containing 85% N_2_, 5% H_2_, and 10% CO_2_ generated using an Anoxomat System (MART Microbiology BV; the Netherlands). *C. difficile* colonies were identified by colonial morphology, Gram stain, fluorescence under UV light, odor, and Vitek MS, and confirmed by the amplification of *tpi* genes by PCR, using primers and amplification procedures previously described (31). All isolates were frozen at −70°C in Brucella broth medium supplemented with 10% glycerol for subsequent analysis.

### RNA extraction for qRT-PCR

*C. difficile* strains were grown in 5 mL of TY broth for 24 h under an anaerobic atmosphere in a jar with an atmosphere containing 85% N_2_, 5% H_2_, and 10% CO_2_ generated using an Anoxomat System (MART Microbiology BV; the Netherlands) at 37°C for 48 h and RNA extracted from the bacterial pellet using the hot phenol method (32). Samples were chilled on ice, centrifuged at 19,000 × *g* for 10 min at 4°C. The aqueous layer was transferred to a 1.5 mL Eppendorf tube, and RNA was precipitated with cold ethanol and incubated at −70°C overnight. The RNA was centrifuged at 19,000 × *g* for 10 min at 4°C. Pellets were washed with cold 70% ethanol and centrifuged at 19,000 × *g* for 5 min at 4°C and air dried for 15 min in the Centrifugal Vacuum Concentrator 5301 (Eppendorf, USA). The quality of RNA was assessed using a NanoDrop (ND-1000; Thermo Scientific, USA). cDNA was synthesized using 1 µg of RNA, 0.2 µg/µL of random hexamer primers, and 2 U/µL of M-MulV-RT (Reverse transcriptase of Moloney Murine Leukemia Virus; Thermo Scientific, USA).

### Expression of toxin genes measured by qRT-PCR

The expression of toxins A, B, and CDT genes was determined by quantitative reverse transcriptase PCR (qRT-PCR) in a LightCycler 480 thermal cycler (Roche Diagnostics,USA). The primers specific for *tcdA*, *tcdB*, and toxin *cdt* genes used are listed in Table 4 (33, 34). Master Mix was prepared forward and reverse primers (20 µM), cDNA (50–100 ng), and SYBR Green Master (Roche Diagnostics, USA) 2×. A 96-multiwell plate containing all samples was loaded into the lightCycler. Each sample was tested in triplicate, and the reported relative expression represents the mean of the three replicates. The expression value of all toxin genes was normalized with the expression of the *C. difficile* housekeeping gene *rpoA* and used to estimate relative expression as follows: relative expression *=* 2^(CTrpoA-CT target gene)^. We selected *C. difficile* 630 and *C. difficile* 027 as control strains (16).

**TABLE 4:** Primers used in this study

Genes	Sequence (5′ to 3′)	Reference
*rpoA*	F-GGATGATATGATGAAGGTTAGAAACCT R-CCCAATCCAAGTTCTTCTAGTTTTTG	Metcalf et al. (33)
*cdtA*	F-TGCAATACTACTTACAAGGCTCCTATAGA R-TCTTTCCCATTCTTTAGCCTTTTC	Stewart and Hegarty (34)
*cdtB*	F-TCAAAATGGATTCACAGCTAATGTAACTACA R-CAGTATTCCATGATTCTCCATTACTATCTTGA	Stewart and Hegarty (34)
*tcdA*	F-GGTAATAATTCAAAAGCGGCT R-AGCATCCGTATTAGCAGGTG	Stewart and Hegarty (34)
*tcdB*	F-GTGTAAGTTTAGGTGCAGCAATCAA R-CCATTATACCTATCTTAGCTTCTATTTCTTGTCT	Stewart and Hegarty (34)

### *In vitro* toxin production and cytotoxin titration assay

All *C. difficile* clinical isolates strains were cultured in Trypticase Yeast Extract broth (TY) and incubated at 37°C for 24–48 h in a jar under an anaerobic atmosphere. Complete culture supernatants were obtained by centrifugation and filter sterilization. Green monkey kidney fibroblast cells (Vero) were grown in 25 cm^2^ flasks with MEM media supplemented with 10% fetal serum (Gibco, USA) and incubated at 37°C in a 5% CO_2_ atmosphere. Aliquots of 200 µL with 5 × 10^3^ cells/mL were distributed per well in 96-well microtiter plates and growth to confluent monolayer. About 10 µL of serial 10-fold dilutions of the test culture-supernatants was applied per well as previously described (6) and incubated for 24 h at 37°C in a 5% CO_2_ atmosphere. Cytotoxic units (CUs) were expressed as the inverse of the maximum dilution that caused rounding of at least 50% of the cells. Positive results were confirmed by neutralization of the cytotoxic effect by mixing 25 µL of a specific polyclonal *C. difficile* antitoxin (Toxin/antitoxin *C. difficile* detection kit; TechLab, Blacksburg, VA 24060, USA) with 25 µL of each supernatant. Each assay was performed by duplicate. *C. difficile* ATCC 9689 was used as positive control.

### Whole-genome sequencing and bioinformatics analysis

*C. difficile* strains were grown overnight in BHI broth, centrifuged, and genomic DNA extracted using the DNeasy Kit (Qiagen, Hilden, Germany). Genome was sequenced using a Hi Seq 2000 (Illumina Inc., San Diego, CA, USA) as described (6). Sequences were assembled *de novo* with default settings with the Shovill pipeline (https://github.com/tseemann/shovill) (35). The contings were annotated using the annotation pipeline Prokka v.1.13 (https://github.com/tseemann/prokka) (36). The 39 *C*. *difficile* genomes were previously sequenced in an earlier study (6). Genome sequences were deposited at the NCBI as part of the 100K Pathogen Genome Project (37) under the BioProject accession number PRJNA203445. Table S1 describes the accession number for each genome sequence.

### Multilocus sequence typing analysis

MLST of all isolates was performed using seven housekeeping genes as previously described (38). The nucleotide sequence of these seven genes (*adk*, *atpA*, *dxr*, *glyA*, *recA*, *sodA*, and *tpi*) (38) was extracted from the sequenced genome of each strain, concatenated and used to assign STs and clades of *C. difficile* according to the PubMLST database using MLST v.2.10. (http://pubmlst.org/cdifficile/) (38).

### Phylogenetic analyses of the whole-genome sequence

A phylogenetic analysis of the 39 strains of *C. difficile* was performed using the complete genome and core-genome sequence to compare the relationship between strains and their evolutionary divergence. Snippy v.2.0 was used to obtain the core single nucleotide polymorphisms. FastTree v.2.1.11 was used to construct the phylogenetic trees using the maximum likelihood method and the Jukes-Cantor evolution model, where the length of the branches representing the genomic distance between the analyzed sequences was obtained. The confidence of the phylogenetic analysis was assessed by bootstrapping with 250 replicates. Phylogenetic trees were edited with iTol v.6.7.5 (https://itol.embl.de/). In addition, other data were added to the respective phylogenetic trees (age, toxin titer, expression, and toxin subtype). The sequence of *C. difficile* ATCC 630 (GenBank AM180355.1) was used as a reference strain.

### Subtyping of TcdA and TcdB

For the identification of toxin variants, the *tcdA* and *tcdB* gene sequences were extracted from pre-computed genome annotations and extracted using probabilistic profile hidden Markov models (profile HMMs) with software HMMER v.3.3.2 (http://hmmer.org). The sequences were aligned with ClustalQ in Seaview v.4, using strain 630 as queries. Sequences were translated from nucleotides to amino acid sequences with Seaview v.4, and the subtype of toxins was determined with DiffBase database (http://diffbase.uwaterloo.ca)(19). The amino acid sequences of *tcdA* and *tcdB* were concatenated, and a phylogenetic tree was built with FastTree v.2.1.11 using the maximum likelihood distances according to the Jones-Taylor-Thorton evolution model and visualized with iTOL. The *P. sordelli* TcsH sequence was used as outgroup for the analysis (16).

### Statistical analyses

The statistical analysis was performed with descriptive analysis, and for the categorical variables, absolute and relative frequencies were obtained, whereas for the continuous variables, the mean was estimated. The proportion test was used to evaluate differences in the frequency of detection of toxin genes and ST between isolates from adults and children. Fisher’s exact test was used to assess differences between TcdA and TcdB subtypes. Fisher’s exact test was used to compare TcdB titer in Vero cells between isolates from adults and children. Fisher’s exact test was calculated for co-morbid conditions between adults and children. A Spearman correlation coefficient was calculated to examine association between toxin subtype and clades. The Wilcoxon rank-sum test assessed differences in toxin gene expression between adult and pediatric strains. Analyzes were performed using Stata version 12 (StatCorporation, College Station, TX, USA).

## SUPPLEMENTAL MATERIAL

Supplemental material spectrum.03947-23-s0001.pdf Table S1.

ASM does not own the copyrights to Supplemental Material that may be linked to, or accessed through, an article. The authors have granted ASM a non-exclusive, world-wide license to publish the Supplemental Material files. Please contact the corresponding author directly for reuse.
