# Supplementary material for: Genomic and phenotypic studies among Clostridioides difficile isolates show a high prevalence of clade 2 and great diversity in clinical isolates from Mexican adults and children with healthcare-associated diarrhea
Source: Microbiol Spectr. 2024 Jun 12;12(7):e03947-23. doi: 10.1128/spectrum.03947-23 (PMC11218462; doi:10.1128/spectrum.03947-23)
Supplement: Supplemental material — Table S1. [file spectrum.03947-23-s0001.pdf]

Supplemental Table 1. NCBI and Short Reads Archives Number of Mexican *C. difficile* Sequences

ID	Adult/Children	Age	Sex	NCBI Number	Short-reads archive Number
**24**	Adult	70	F	SAMN21923162	SRR17242259
**29**	Adult	69	M	SAMN21923164	SRR17242237
**36**	Adult	60	M	SAMN21923165	SRR17242226
**37**	Adult	24	F	SAMN21923166	SRR17242215
**38**	Adult	50	M	SAMN21923167	SRR17242204
**47**	Adult	58	F	SAMN21923169	SRR17242182
**78**	Adult	60	F	SAMN21923172	SRR17242267
**82**	Adult	57	F	SAMN21923173	SRR17242266
**91**	Adult	81	F	SAMN21923175	SRR17242264
**103**	Adult	81	M	SAMN21923177	SRR17242262
**111**	Adult	66	F	SAMN21923180	SRR17242258
**128**	Adult	48	M	SAMN21923181	SRR17242257
**129**	Adult	70	M	SAMN21923182	SRR17242256
**138**	Adult	46	F	SAMN21923183	SRR17242255
**140**	Children	3	F	SAMN21923184	SRR17242254
**144**	Adult	60	F	SAMN21923185	SRR17242253
**146**	Adult	67	F	SAMN21923186	SRR17242252
**148**	Children	13	F	SAMN21923187	SRR17242251
**160**	Adult	65	F	SAMN21923188	SRR17242250
**166**	Adult	37	M	SAMN21923189	SRR17242249
**167**	Adult	51	M	SAMN21923190	SRR17242247
**171**	Adult	75	F	SAMN21923191	SRR17242246
**174**	Adult	49	F	SAMN21923192	SRR17242245
**186**	Adult	65	F	SAMN21923194	SRR17242243
**197**	Adult	26	F	SAMN21923196	SRR17242242
**253**	Children	3	F	SAMN21923199	SRR17242239
**267**	Children	15	M	SAMN21923200	SRR17242238
**268**	Children	15	M	SAMN21923201	SRR17242236
**271**	Children	0.7	F	SAMN21923202	SRR17242235
**281**	Adult	66	F	SAMN21923204	SRR17242233
**291**	Children	6	M	SAMN21923206	SRR17242231
**295**	Adult	76	F	SAMN21923207	SRR17242230
**299**	Adult	51	F	SAMN21923208	SRR17242229
**300**	Adult	63	M	SAMN21923209	SRR17247447
**322**	Adult	72	F	SAMN21923210	SRR17242228
**336**	Adult	35	F	SAMN21923212	SRR17242225
**361**	Adult	66	M	SAMN21923213	SRR17242224
**365**	Adult	82	M	SAMN21923214	SRR17242223
**375**	Children	14	M	SAMN21923216	SRR17242221
